# Enhancement and quenching of ZnO nanowire luminescence by electron irradiation

**DOI:** 10.1186/s11671-025-04352-1

**Published:** 2025-09-12

**Authors:** Edwin Eobaldt, Maximilian Zapf, Gesine Wolf, Sam Imani, Johannes Nicklaus, Sven Schönherr, Carsten Ronning

**Affiliations:** Institute of Solid State Physics, Friedrich Schiller University Jena, 07743 Jena, Germany

**Keywords:** Semiconductor nanowires, Electron irradiation, Optical properties

## Abstract

Electron microscopy is an essential tool in the fabrication and characterization of optoelectronic devices. This study systematically investigated the stability of zinc oxide nanowires under electron irradiation, focusing on spontaneous and stimulated emission as well as their waveguiding properties. Spontaneous emission initially increases, which we attribute to the desorption of surface species, leading to a reduced surface band bending and enhanced luminescence. However, continued electron irradiation results in hydrocarbon formation on the nanowire surface, increasing waveguiding losses. These increased optical losses, in turn, raise the lasing threshold of nanowire lasers. Our findings offer valuable insights into the electron imaging parameters for optimizing semiconductor nanowires in optoelectronic applications.

## Introduction

High-resolution imaging techniques, such as scanning electron microscopy (SEM) [1] and transmission electron microscopy (TEM) [2, 3], have become indispensable tools for characterizing nanoscale materials. These techniques allow probing structural and compositional details of nanomaterials, providing critical insights that drive advances in nanotechnology. The importance of these tools has been highlighted by recent advances in nanorobotic manipulation, which facilitate direct nanoscale characterization and assembly [4]. However, the actual electron beams that enable such detailed imaging, well below the Abbe-limit, can inadvertently alter the optical properties of the studied materials. Researchers first observed luminescence quenching during the acquisition of cathodoluminescence (CL) spectra of semiconductors, indicating a detrimental impact of electron irradiation. In ZnO bulk crystals, prolonged electron irradiation led to a notable reduction in CL intensity on the Zn-terminated surface, whereas the O-terminated surface initially exhibits a slight enhancement in CL, followed by a subsequent decline [5]. In GaN nanowire (NW) ensembles, a pronounced luminescence quenching was observed under electron irradiation indicating a relation to the greatly enhanced surface-to-volume ratio in NWs, compromising their performance in practical optoelectronic applications [6].

ZnO NWs have emerged as a cornerstone in the development of advanced optoelectronic devices, owing to their exceptional physical properties, including a wide direct bandgap (3.37 eV) and a large exciton binding energy (60 meV) allowing for stable operation at room temperature. Further, ZnO NWs exhibit a compelling property in their ability to achieve lasing without the need of external cavities [7]. These structures show intrinsic optical gain when subjected to adequately high pumping conditions. Their morphology and the pronounced refractive index contrast to the surrounding enable them to function effectively as waveguides and optical resonators [8], facilitating efficient light confinement and lasing action [9]. These characteristics make ZnO NWs ideal candidates for applications such as ultraviolet light-emitting diodes [10, 11], laser diodes, and photodetectors [12, 13], where efficient and stable luminescence is paramount.

However, the effectiveness of these applications depends on preserving the pristine luminescence properties of ZnO NWs, which are particularly susceptible to degradation under electron beam exposure. The exposure may introduce point defects, which can significantly alter the material’s optical properties [14]. Furthermore, electron irradiation has been shown to transform the surface properties of ZnO NWs, shifting them from a hydrophilic to a hydrophobic state [15].

Despite the widespread use of electron microscopy in nanomaterials research, there is still a lack of comprehensive understanding of how electron irradiation impacts the luminescence of ZnO NWs. While the effects of electron irradiation on bulk semiconductors and thin films have been relatively well studied [16, 17], the unique characteristics of semiconducting NWs with its high surface-to-volume ratio necessitate a focused investigation.

This study seeks to address this gap by systematically investigating the influence of electron irradiation on the luminescence and lasing properties of ZnO NWs. By using a combination of CL and photoluminescence (PL) measurements, this study aims to quantify the changes in spontaneous and stimulated emission regimes and elucidate the mechanisms underlying these changes.

## Experimental

Zinc oxide NWs were grown in a horizontal three-zone tube furnace by a vapor transport technique [18]. As the source material, a mixture of pure ZnO powder and graphite (mass ratio 7:1) was used and sublimated at 1050 °C in the middle zone of the furnace. Thereafter, the gaseous source material was transported towards Au-coated (thin film of ~ 10 nm) silicon substrates with the help of a carrier gas containing Ar and O_2_ (each 10 sccm). The growth lasted 60 min at a pressure of 7 mbar. Subsequently, ZnO nanowires were transferred from the growth substrate by imprint to a clean SiO_2_/Si substrate, resulting in dispersed single nanowires for the optical measurements. In this study, we selected only ZnO nanowires with diameters of ~ 200 nm for all measurements.

The NWs were irradiated with the electron beams of a JEOL-6490 SEM with a LaB_6_ electron gun (maximum energy 30 keV, maximum current 100 nA, lateral resolution ~ 10 nm) and a FEI DualBeam Helios NanoLab 600i system (maximum energy 30 keV, maximum current 22 nA, lateral resolution ~ 1 nm).

The CL measurements were performed with a GATAN MonoCL3 + recording system within the JEOL-6490 SEM. The NW emission was collected by a parabolic mirror during focused electron beam excitation and directed into a 300 mm Czerny-Turner type monochromator. Here, a 1200 grooves/mm grating was used to disperse the light which was subsequently detected by a Peltier-cooled photomultiplier (Hamamatsu R943). The spectra were acquired serially with step sizes of 1–5 nm.

The optical characterization of the NWs was done by micro-photoluminescence (µPL) measurements. Hereby, the spot of a continuous-wave HeCd laser (325 nm) was focused to a diameter of ~ 1 μm with a 50× UV objective NA = 0.42 to allow for a spatially defined excitation. The incident laser power could be adjusted by several orders of magnitudes by using neutral density filters in the beamline, while a silicon photodiode measured the power in situ. The emitted luminescence was collected perpendicular to the substrate plane by the same objective and subsequently focused into a 500 mm monochromator (Princeton Instruments SP-2500i). After passing a 150 grooves/mm (resolution ~ 0.8 nm) grating, the dispersed light is detected by a liquid-nitrogen cooled, front-illuminated CCD camera.

For the NW lasing measurements, the third harmonic of a Nd: YAG laser (355 nm, 5 mJ, 100 Hz, 7ns) within the same setup was used for excitation. Contrary to above, the laser spot was intentionally defocused to ~ 20 μm to ensure a full illumination of the NWs. In addition, a 50× NUV objective (NA = 0.43) and a precise grating with 1200 grooves/mm (resolution ~ 0.1 nm) were used.

**Fig. 1:**
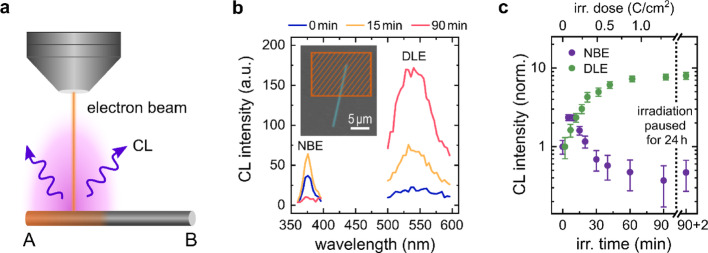
Electron irradiation and cathodoluminescence of single ZnO nanowires. **a** The electron beam was rastered along roughly half of the NW (end A), and the other half remained pristine and served as a reference (end B). **b** CL spectra obtained from the region indicated in the inset SEM image of a ZnO nanowire with a diameter of ~ 200 nm and length ~ 16 μm. The NBE and DLE spectra were acquired successively. **c** Integrated CL intensity as a function of electron irradiation for the NBE (purple) and the DLE (green). After an electron irradiation time of 90 min the irradiation was paused for 24 h

## Results and discussion

First, the electron beam of an SEM was scanned across half of a single NW lying on a silica substrate while the CL signal was acquired, as schematically shown in Fig. 1a and the inset in Fig. 1b. The excitonic recombination of ZnO (NBE, near band edge) at ~ 380 nm and the defect recombination (DLE) centered at ~ 550 nm were recorded separately such that the data could be taken within 60 s. Thus, electron beam-induced changes during the acquisition can be neglected. The CL spectra of DLE and NBE are plotted in Fig. 1b, which were alternately measured in succession for a total time of 90 min at 10 kV and an electron flux density of ~ 0.24^− 1^ cm^− 2^. Figure 1c shows the CL intensity of NBE and DLE as a function of irradiation time (and fluence/dose). Significant differences are evident here: While the DLE signal increases monotonically and saturates for prolonged irradiation, the NBE signal increases for the first ~ 10 min of irradiation until it starts decreasing and finally saturates well below its initial value. In the following, the irradiation was paused for 24 h and the sample was kept in the evacuated SEM chamber. After pausing, spectra were recorded again, which did not show a change in the signals. Thus, charging effects can be neglected as they would lead to a significant jump in the CL signal when continuing the irradiation [19]. Remarkably, also pausing the irradiation and keeping the samples in air did hardly show any effects on the curve progression. After irradiating pristine nanowires for 10 min, the irradiation was paused for different times (10 min–2 h, respectively). When continuing the irradiation, the curve continued undisturbed. Moving the sample out of the vacuum and keeping it in air for similar time spans also showed no changes.

It is unlikely that defect creation can account for the observed trends in NBE and DLE, given that the reported atomic displacement thresholds are in the range of 300 keV for the electron energy [20, 21], which is well below the acceleration voltage of 10 keV that has been used in this study. Instead, the initial increase in the NBE may be attributed to the electron-beam-induced desorption of surface species. Due to the permanent presence of surface adsorbates (primarily O_2_) [22, 23], incident electrons can stimulate these adsorbates to either desorb directly or via recombination with secondary holes [12]. This leads to a reduction of the band bending at the surface, which in turn leads to a higher rate of radiative recombination. In that way, the initial increase of the NBE, as observed in Fig. 1c, can be explained. Surface desorption processes also offer an explanation to the increase in DLE. When assuming oxygen vacancies at the surface V_O_ as the key recombination channel for the DLE, its increase can be explained by the electron beam induced activation of surface V_O_ that were previously passivated by surface adsorbates.

A prolonged electron irradiation has detrimental effects, as clearly seen for both the NBE and DLE luminescence in Fig. 1c. Already studied in the 1960s for cathode ray tubes in TVs [24], the degradation of luminescent phosphors was ascribed to electron-stimulated surface chemical reactions, primarily involving carbon [25]. It was observed that carbon layers of more than 100 nm thickness built up that led to an attenuation of both, the impact of the incident electron beam and the cathodoluminescence (CL) signal [26]. Such a drastic effect, however, can be ruled out for our experiments, since they require high concentration of hydrocarbons in the residual vacuum. On the other hand, hydrocarbon molecules from the nanowire surface or vacuum chamber can still be cracked by the electron beam in modern SEMs, forming a thin carbonaceous layer. Secondary electrons can partially counteract this build-up through carbon erosion, influenced by temperature, acceleration voltage, and beam current [27]. Prolonged electron beam exposure can reduce surface carbon due to beam-induced chemical reactions, which may create volatile species or cause permanent surface changes [28]. These reactions are affected by vacuum pressure and residual gas composition. Nanowires, with their high surface-to-volume ratio, are particularly susceptible to such effects. For GaN nanowires, even a sub-monolayer of carbon can reduce luminescence by two orders of magnitude [6]. This reduction is not attributed to an electron or optical attenuation but to enhanced non-radiative surface recombination caused by carbon adatoms.

**Fig. 2:**
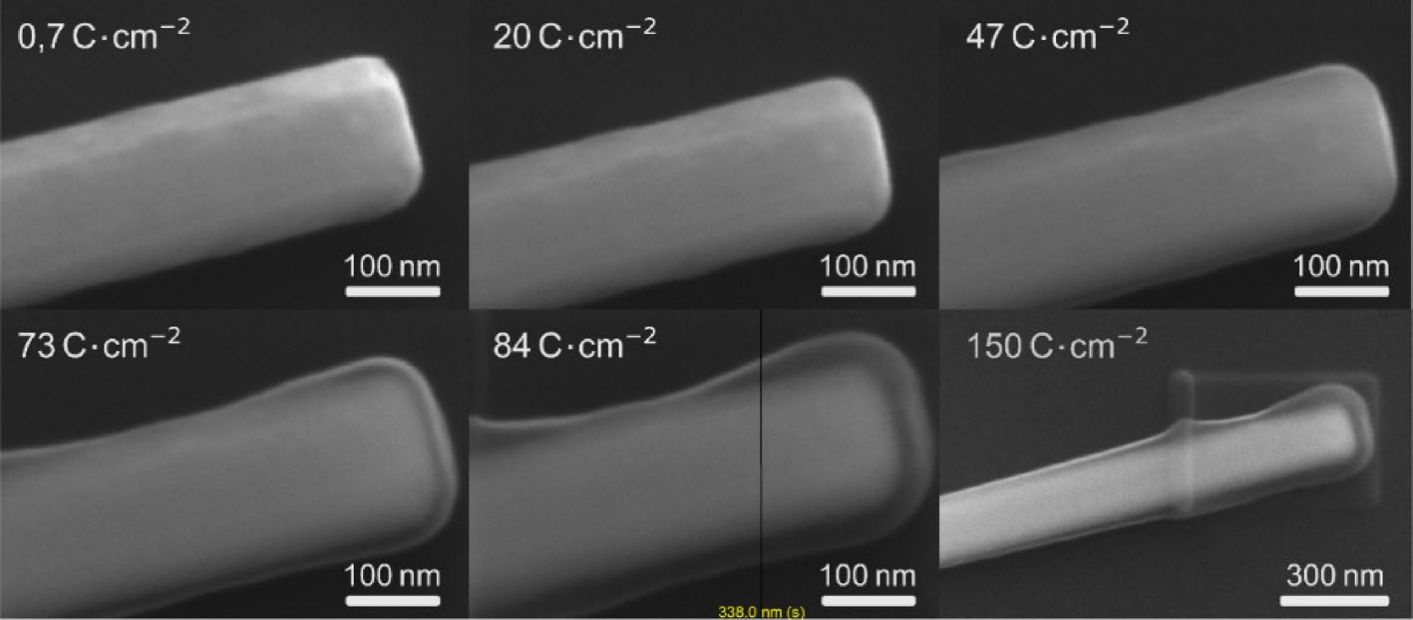
Sanning electron microscope (SEM) images of the facet end of one single ZnO NWs, which was electron irradiated with an average electron flux of about 37 mCcm^− 2^s^− 1^ as a function of irradiation fluence

We investigated this hypothesis in more detail by exposing ZnO NWs with extremely high electron fluences up to 150 Ccm^− 2^, which are unreasonable and never needed in imaging. This could be only achieved by irradiating only one small part of the NW. Indeed, even under high vacuum conditions, an increased thickness of the NW could be observed in the SEM images shown in Fig. 2 as a function of electron irradiation fluence. The SEM of the NWs exposed to the highest irradiation fluences show furthermore an evolving coating layer with different contrasts. Even though detailed chemical analysis was not possible, such as by EDS, it is likely that the layer contains mainly out of carbon originating from cracking of hydrocarbon molecules from the residual gas by the electron beam.

**Fig. 3:**
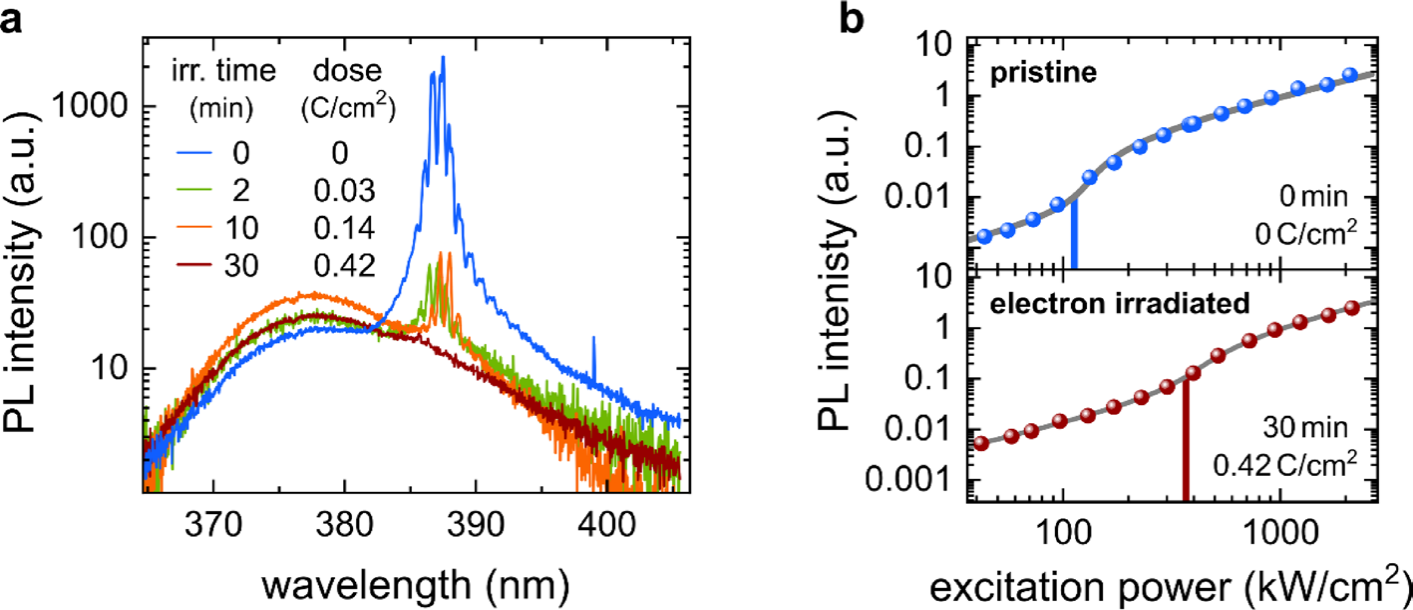
**a** Lasing spectra of the ZnO nanowire laser prior and after electron irradiation as a function of irradiation fluence (dose), all taken at a similar excitation intensity of ~ 240 kWcm^− 2^, which is about two times the laser threshold of the pristine nanowire. The electron irradiation induced a strong quenching of the stimulated emission at this excitation intensity. **b** Excitation power dependency (light-in light-out curve) prior and after electron irradiation. The lasing threshold, which was estimated by a multimode laser model (solid lines) [30], increased threefold after the electron irradiation

Next, the impact of electron irradiation on the stimulated emission of ZnO NWs was investigated. To this end, first, a PL power series was measured for several individual NWs, i.e., measurements were acquired over a wide range of excitation powers. The excitation power, in turn, was kept well below the degradation threshold such that we can rule out laser-induced damaging of the NW [29]. Afterwards, the whole NW was irradiated by the SEM electron beam, and another PL power series was recorded. This procedure was repeated to monitor changes in the NW laser output. In order to maintain similar excitation conditions for the successive optical measurements, the alignment of the PL setup and the orientation of the NW in respect to the laser beam were kept constant. Figure 3a shows the lasing spectra of a single NW for different irradiation times at a fixed excitation power (twice the initial lasing threshold intensity). A drastic quenching of the lasing modes is observed with increasing irradiation time. After 30 min (~ 0.42 Ccm^− 2^), the Fabry-Pérot modes have almost completely disappeared (dark red line). In contrast to the CL measurements, no initial increase can be observed. It is notable that there is also no significant alteration in the mode spacing, which suggests that neither the effective resonator length nor the effective refractive index of the NW underwent a significant change [7]. In addition, the lasing threshold was estimated for each measurement by applying a multimode laser model [30] (solid lines) to the light-in light-out curve of the NW laser, as depicted in Fig. 3b. The strong effect of electron irradiation is also evident here: the lasing threshold increases threefold.

**Fig. 4:**
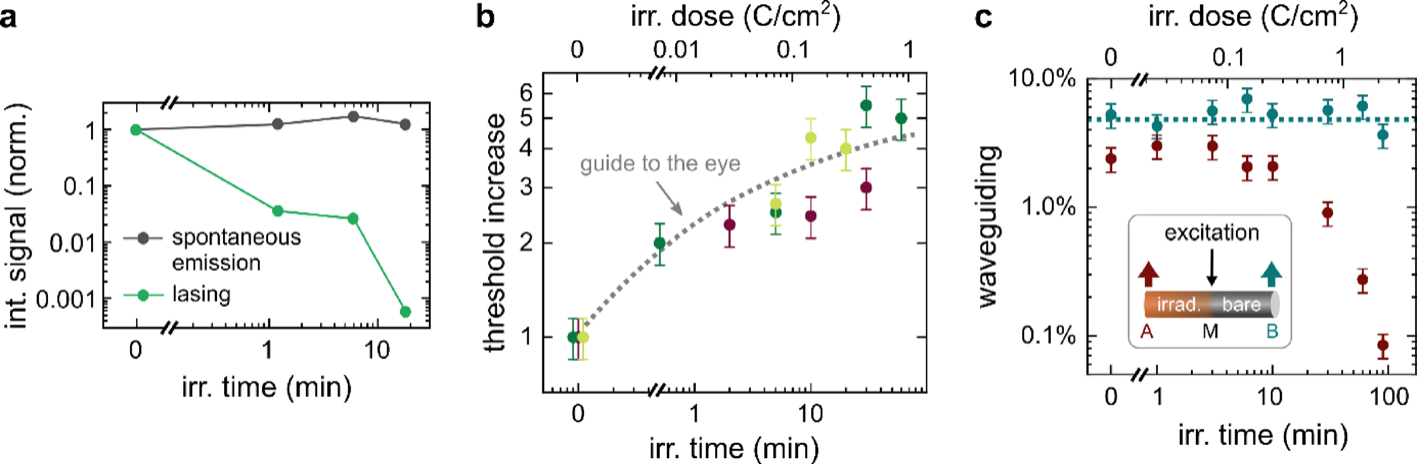
**a** Integrated signal from the spontaneous emission (gray) and the NW lasing modes (green). The stimulated emission drastically decreases with electron irradiation while the spontaneous emission stays almost constant. **b** Threshold increase as a function of electron irradiation for 3 different NWs. **c** Electron irradiation dependence of the waveguided NBE intensity (red dots) of a ZnO nanowire with a length of ~ 26 μm and a diameter of ~ 200 nm. As a reference (blue), the waveguiding through the pristine part of the NW is added

Integrated PL signals of the laser modes and the spontaneous emission background are plotted in Fig. 4a as a function of irradiation time on a double-logarithmic scale. Consistent with the CL measurements, the spontaneous emission first increases slightly and then decreases. In stark contrast, the signal of the laser modes falls by more than 3 orders of magnitude after just 20 min of electron irradiation. To further analyse the increase of the lasing threshold as a function of irradiation time, several NWs were irradiated and measured stepwise. Figure 4b shows the resulting plot: an increase in the lasing threshold can be seen for all NWs. The effect is particularly large in the first exposure step, demonstrating that even small electron beam exposure times and fluences have a strong detrimental impact on the performance of NW lasers. With further electron irradiation the increase in lasing threshold weakens until it saturates for high fluences. In total, the threshold increases to three to six times the initial value of the pristine NW.

Unlike spontaneous emission, where light is emitted randomly, lasing requires light to be guided through the NW and reflected at the end facets such that the net gain exceeds the losses. A possible explanation for the increasing threshold with continued irradiation could thus involve increased waveguiding losses. To investigate this, waveguiding experiments were performed on a single nanowire laser, where approximately half of it was irradiated with electrons.

The used PL setup made it possible to decouple the excitation laser spot from the PL collection spot. As shown in the inset of Fig. 4c, the NW was excited at its middle position (M) and the PL was collected at the facet ends A (irradiated part) and B (pristine part). After the reference measurement, approximately 40% of the NW was irradiated, ensuring that spot M remained pristine. Waveguiding in the NW occurs through an absorption-emission-absorption process of exciton-polaritons [31, 32]. Figure 4c depicts the waveguiding transmission efficiency as a function of irradiation time on a double-logarithmic scale. The data were derived from the NBE intensity at the NW ends, which were normalized to the corresponding signal at the excitation spot (I_A_/I_M_ and I_B_/I_M_). As expected, the waveguiding efficiency that was guided through the pristine part (light green data points) remains constant. On the contrary, light guided through the electron-irradiated part (red data points) exhibits a slight increase for low doses, followed by a significant decline of more than one order of magnitude for higher doses. The initial increase in waveguiding efficiency might be again attributed to surface desorption and the subsequent reduction of non-radiative surface recombination. With prolonged electron irradiation, the signal of the exposed part drastically reduces, indicating increased waveguiding losses. These findings are in good agreement with the lasing measurements, where the lasing threshold was more than twice as high for similar electron fluences of ~ 0.1 Ccm⁻². Thus, enhanced waveguiding losses are a significant factor contributing to the pronounced increase in lasing threshold observed following electron irradiation. This can be attributed to the formation of non-radiative surface defects due to electron beam-induced chemical surface reactions [6], as discussed already before. An electron beam-induced carbonaceous surface layer is the likely cause of luminescence quenching due to the formation of non-radiative recombination centers at the surface.

## Conclusion

The stability of ZnO nanowires under electron irradiation was systematically studied, focusing on spontaneous and stimulated emission as well as waveguiding properties. Measurements were carried out before and after exposure to various electron beam irradiation fluences and durations. An initial increase in spontaneous emission was observed, attributed to the desorption of surface species that reduced surface band bending and, consequently, enhanced luminescence. However, this process noticeably slowed down with prolonged electron irradiation, very likely due to hydrocarbon formation on the nanowire surface induced by chemical reactions triggered by the electron beam. As irradiation further continued, spontaneous emission showed a slight decrease. Nevertheless, the most pronounced effect of electron irradiation was on the waveguiding properties, with a substantial reduction in the amount of light guided through the nanowire as exposure progressed. Moreover, stimulated emission also experienced a significant decline, primarily due to the activation of non-radiative recombination processes triggered by the electron irradiation. This increase in non-radiative recombination significantly suppressed the active waveguiding process, resulting in elevated optical losses and a much higher lasing threshold in nanowire lasers. Future studies should include measurements of emission dynamics, such as with time-resolved PL or pump-probe experiments, to clearly distinguish between changes in radiative versus non-radiative recombination.

Nevertheless, our findings highlight the importance of controlling the vacuum environment during electron microscopy to minimize hydrocarbon contamination, such as by using oil-free pumps. In addition, the electron dose must be limited when inspecting nanoscale optoelectronic devices by SEM to avoid surface reactions that are crucial for nanoscale devices with their high surface-to-volume ratio. This is not only true for the ZnO model material that we used in this study but is also likely the case for other (nanowire) materials (e.g., GaN, In₂O₃, Ga₂O₃, SiC). We further showed that semiconductor NWs are ideal tools for studying electron-beam induced effects given their quasi-1D structure. Finally, these insights are crucial for optimized processing of nanoscale semiconductors for optoelectronic applications, where electron beam exposure is often unavoidable. In fact, in-line SEM systems are used in semiconductor fab lines as “nondestructive” imaging tools to determine critical dimensions.

## Data Availability

Data is provided within the manuscript. Original files can be provided upon reasonable request.
